# Machine-learning approach identifies a pattern of gene expression in peripheral blood that can accurately detect ischaemic stroke

**DOI:** 10.1038/npjgenmed.2016.38

**Published:** 2016-11-30

**Authors:** Grant C O’Connell, Ashley B Petrone, Madison B Treadway, Connie S Tennant, Noelle Lucke-Wold, Paul D Chantler, Taura L Barr

**Affiliations:** 1 Center for Basic and Translational Stroke Research, Robert C. Byrd Health Sciences Center, West Virginia University, Morgantown, WV, USA; 2 Department of Pharmaceutical Sciences, School of Pharmacy, West Virginia University, Morgantown, WV, USA; 3 Department of Biology, Eberly College of Arts and Sciences, West Virginia University, Morgantown, WV, USA; 4 Center for Cardiovascular and Respiratory Sciences, Robert C. Byrd Health Sciences Center, West Virginia University, Morgantown, WV, USA; 5 Division of Exercise Physiology, School of Medicine, West Virginia University, Morgantown, WV, USA; 6 CereDx Incorporated, Morgantown, WV, USA

## Abstract

Early and accurate diagnosis of stroke improves the probability of positive outcome. The objective of this study was to identify a pattern of gene expression in peripheral blood that could potentially be optimised to expedite the diagnosis of acute ischaemic stroke (AIS). A discovery cohort was recruited consisting of 39 AIS patients and 24 neurologically asymptomatic controls. Peripheral blood was sampled at emergency department admission, and genome-wide expression profiling was performed via microarray. A machine-learning technique known as genetic algorithm k-nearest neighbours (GA/kNN) was then used to identify a pattern of gene expression that could optimally discriminate between groups. This pattern of expression was then assessed via qRT-PCR in an independent validation cohort, where it was evaluated for its ability to discriminate between an additional 39 AIS patients and 30 neurologically asymptomatic controls, as well as 20 acute stroke mimics. GA/kNN identified 10 genes (*ANTXR2*, *STK3*, *PDK4*, *CD163*, *MAL*, *GRAP*, *ID3*, *CTSZ*, *KIF1B* and *PLXDC2*) whose coordinate pattern of expression was able to identify 98.4% of discovery cohort subjects correctly (97.4% sensitive, 100% specific). In the validation cohort, the expression levels of the same 10 genes were able to identify 95.6% of subjects correctly when comparing AIS patients to asymptomatic controls (92.3% sensitive, 100% specific), and 94.9% of subjects correctly when comparing AIS patients with stroke mimics (97.4% sensitive, 90.0% specific). The transcriptional pattern identified in this study shows strong diagnostic potential, and warrants further evaluation to determine its true clinical efficacy.

## Introduction

Stroke is currently the leading cause of disability and the fifth leading cause of death in the United States.^1^ It is well established that early and accurate diagnosis improves outcome by increasing the probability of successful intervention;^2,3^ however, the diagnostic tools currently available to clinicians for the identification of stroke have significant limitations.

Although neuroradiological imaging is the gold standard for diagnosis of stroke,^4^ it is inaccessible in the field and at the initial point of contact in emergency departments. Furthermore, such imaging techniques are often not immediately available in hospitals without dedicated stroke centres, such as smaller facilities and those which serve rural areas.^5^ As a result, crucial decisions regarding the triage of potential strokes by emergency department staff and emergency medical technicians are based on the assessment of overt patient symptoms using stroke recognition and severity scales such as the Cincinnati pre-hospital stroke scale (CPSS) and the National Institutes of Health stroke scale (NIHSS).^4^ In the hospital setting, the ability to identify stroke with such assessments is highly inconsistent, with an estimated sensitivity ranging from 44 to 85%, and specificity ranging from 64 and 98%.^6^ The sensitivity and specificity of these assessments are even lower in the pre-hospital setting,^7^ where the ability to quickly identify stroke facilitates the transfer of patients to stroke-ready hospitals, increasing the chances of appropriate treatment and positive outcome.^8^ Due to these current limitations, a rapidly measurable blood-based biomarker panel could be invaluable in informing pre-hospital and in-hospital decisions early in the acute phase of care, and could ultimately expedite access to interventional treatment.^9^

As a result, there has been a substantial push for the identification of stroke-associated peripheral blood biomarkers. The earliest stroke biomarker studies focused on the peripheral blood proteome, and countless protein-based biomarker panels have been evaluated to date. While a handful of these protein-based panels have demonstrated a strong ability to differentiate between stroke patients and healthy controls lacking the presence of cardiovascular disease (CVD) risk factors, a majority have failed to achieve specificities and sensitivities approaching 90% when tested against clinically relevant control groups.^9–13^ More recently, the peripheral blood transcriptome has emerged as a potential source of stroke biomarkers, as preliminary reports have suggested that gene expression in the peripheral immune system is highly responsive to ischaemic brain injury.^14–16^ Most notably, Tang *et al.* identified a panel of 18 genes whose expression levels demonstrated the ability to discriminate between acute ischaemic stroke patients (AIS) and healthy controls with 93.5% sensitivity and 89.5% specificity using combined expression data generated from three blood draws obtained over the first 24 h of hospitalisation.^16,17^ While the necessity to obtain multiple blood samples limited this biomarker panel with regards to acute stroke triage, this work provided proof of principle that stroke-induced transcriptional changes in the peripheral immune system could be used to identify stroke with relatively high levels of accuracy. Thus, it is plausible that implementation of a robust biomarker discovery approach could identify transcriptional stroke markers with the potential to be diagnostically useful during the acute phase of care.

Analysis of high-dimensional gene expression data using a pattern-recognition approach known as genetic algorithm k-nearest neighbours (GA/kNN) has been successfully used in a small number of cancer studies to identify diagnostically relevant biomarker panels with strong discriminatory ability.^18–20^ The GA/kNN approach combines a powerful search heuristic, GA, with a non-parametric classification method, kNN. In GA/kNN analysis, a small combination of genes (referred to as a chromosome) is generated by random selection from the total pool of gene expression data (Supplementary Figure 1A). The ability of this randomly generated chromosome to discriminate between sample classes is then evaluated using kNN. In this evaluation, each sample is plotted as a vector in a multidimensional feature space where the coordinates of the vector comprises the expression levels of the genes of the chromosome. The class of each sample is then predicted based on the majority class of the nearest neighbours, or other samples that lie closest in Euclidian distance within the feature space (Supplementary Figure 1B). The ability of the chromosome to discriminate between classes is quantified as a fitness score, or the proportion of samples which the chromosome is correctly able to classify. A termination cutoff (minimum proportion of correct classifications) determines the level of fitness required to pass evaluation. A chromosome which passes kNN evaluation is labelled as a near-optimal solution and recorded, while a chromosome which fails undergoes repeated cycles of mutation and re-evaluation until a near-optimal solution is reached (Supplementary Figure 1A). This entire search paradigm is performed multiple times (typically hundreds of thousands) to generate a heterogeneous pool of near-optimal solutions (Supplementary Figure 1C). The discriminatory ability of each gene is then ranked according to the number of times it appears in the near-optimal solution pool (Supplementary Figure 1D), and the collective discriminatory ability of the top-ranked genes can then be tested via kNN in a leave-one-out cross-validation (Supplementary Figure 1E). This approach has been utilised to generate biomarker panels capable of optimally discriminating between cancerous and non-cancerous colon biopsies,^20^ primary and metastatic melanoma tumours,^18^ as well as between B-cell lymphoma sub-types,^19^ all with accuracies ranging between 95 and 100%.

While GA/kNN has proven robust in several applications in the field of cancer, it has yet to be utilised for biomarker discovery in the realm of cardiovascular disease (CVD). In this study, we applied the GA/kNN approach to analyse peripheral blood gene expression data generated via microarray to identify transcriptional patterns which could potentially be optimised for the detection of AIS in the acute phase of care.

## Results

### Discovery cohort

In order to identify potential transcriptional biomarkers for the identification of AIS, we first recruited a discovery cohort consisting of 39 AIS patients and 24 neurologically asymptomatic controls. In terms of demographic and clinical characteristics, AIS patients were older than controls, and displayed a higher prevalence of CVD risk factors such as hypertension and dyslipidaemia (Table 1). Furthermore, AIS patients displayed a more substantial history of cardiac conditions such as myocardial infarction and atrial fibrillation, and higher proportion of AIS patients reported as currently taking antihypertensives and anticoagulants.

**Figure 1:**
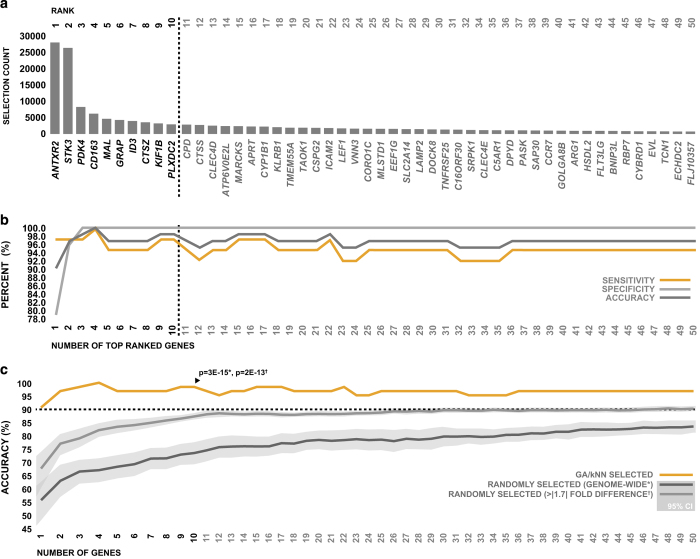
Top 50 genes selected by GA/kNN for identification of AIS. (**a**) The top 50 peripheral blood transcripts ranked by GA/kNN based on their ability to discriminate between AIS patients and neurologically asymptomatic controls in the discovery cohort. (**b**) Combined ability of the expression levels of top 50 genes selected by GA/kNN to discriminate between AIS patients and neurologically asymptomatic controls in the discovery cohort using kNN. (**c**) Ability of the expression levels of the top 50 genes selected by GA/kNN to discriminate between neurologically asymptomatic controls and AIS patients via kNN compared with the expression levels of genes selected at random. The accuracy of the top 10 genes selected by GA/kNN was specifically tested against the accuracy of randomly selected genes using single sample two-way *t*-test.

Peripheral whole blood was sampled from patients at emergency department admission, and genome-wide expression profiling was performed via microarray. Gene expression data were subjected to GA/kNN analysis, and genes were ranked based on the ability of their expression levels to discriminate between AIS patients and controls, according to the number of times they were selected as part of a near-optimal solution (Figure 1a). The expression levels of top 50 genes identified by GA/kNN displayed a strong ability to discriminate between groups using kNN in leave-one-out cross-validation; a combination of just the top 10 ranking genes (*ANTXR2, STK3, PDK4, CD163, MAL, GRAP, ID3, CTSZ, KIF1B* and *PLXDC2*) were able to classify 98.4% of subjects in the discovery cohort correctly with a sensitivity of 97.4% and specificity of 100% (Figure 1b).

In order to evaluate the robustness of our GA/kNN analysis in terms of its ability to select optimally discriminative genes, we compared the ability of the expression levels of top 50 genes selected by GA/kNN to differentiate between stroke patients and controls to that of genes selected at random. Specifically, we compared the accuracy of GA/kNN-selected genes to the accuracy of 50 sets of 50 genes randomly generated from the total pool of gene expression data, as well as to the accuracy of 50 sets of 50 genes randomly selected from a subpool of genes that displayed greater than 1.7-fold differential regulation between groups. The top genes selected by GA/kNN performed significantly better than genes selected at random genome wide, as well as significantly better than genes selected at random from those which were differentially regulated greater than 1.7-fold (Figure 1c). Collectively, the results of this analysis, in combination with the levels of accuracy observed, suggest that our biomarker discovery strategy was effective at selecting genes with optimal diagnostic potential in terms of the subjects of the discovery cohort. Because the use of genes beyond the top 10 did not appear to improve overall accuracy (Figure 1b), and displayed diminishing diagnostic robustness relative to genes selected at random (Figure 1c), we chose to focus on only the top 10 genes for the remainder of our analysis.

**Figure 2:**
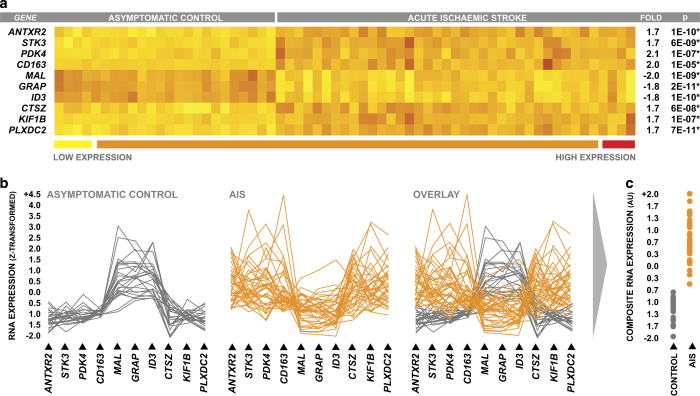
Differential expression of top-ranked genes within the discovery cohort. (**a**) Peripheral blood differential expression of the top 10 genes selected by GA/kNN in discovery cohort neurologically asymptomatic controls and AIS patients, with fold changes reported relative to control. Statistical significance of intergroup differences in gene expression was determined via two-sample two-way *t*-test, and *P*-values were corrected to account for multiple comparisons via Holm's Bonferroni method. (**b**) Coordinate pattern of peripheral blood expression across the top 10 genes plotted for individual subjects in both experimental groups. (**c**) Composite RNA expression levels of the top 10 genes generated via principal components analysis.

When comparing the peripheral blood expression levels of the top 10 genes between AIS patients and controls, the magnitude of differential expression was modest in terms of fold change in the case of most genes; however, differences in expression levels between groups were highly consistent across all subjects, which was reflected by high levels of statistical significance in parametric statistical testing (Figure 2a). The combined discriminatory power of the top 10 genes was evident when their coordinate expression levels were plotted on a continuum for each individual subject; the overall pattern of expression was strikingly different between AIS patients and controls, and it was clear that the overall pattern of expression was more diagnostically powerful than the expression levels of any given gene on its own (Figure 2b).

In order to more intuitively explore the relationship between the pattern of gene expression observed across the top 10 genes and relevant clinical characteristics, we first used principal components analysis to describe the expression levels of the top 10 genes as single composite RNA expression variable. The expression levels of the top 10 genes were highly correlated, and a single principal component was able to describe 70% of the collective variance in expression (Supplementary Table 1A). The result component scores (composite RNA expression) were strongly correlated with the expression levels of each of the individual candidate gene (Supplementary Table 1B), and visually appeared to summarise the gene expression pattern well (Figure 2c).

**Figure 3:**
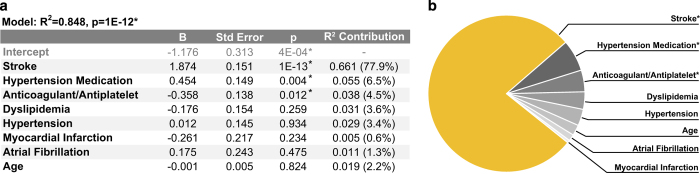
Influence of potentially confounding clinical and demographic characteristics on the expression levels of the top 10 genes. (**a**) Multiple regression model generated by regressing potentially confounding clinical and demographic characteristics against the composite RNA expression levels of the top 10 genes selected by GA/kNN in the discovery cohort. (**b**) Graphical representation of the relative contribution of each regressor towards the total variance in composite RNA expression explained by the model.

We first used this composite RNA expression variable to examine the influence of potentially confounding intergroup differences in clinical and demographic characteristics on the expression levels of the top 10 genes. Stroke, age, anticoagulant status, hypertension, antihypertensive status, dyslipidaemia, history of myocardial infarction and history of atrial fibrillation were regressed against the composite RNA expression levels of the top 10 genes using multiple regression. We then performed variance decomposition via the Lindeman-Merenda-Gold (LMG) method to estimate the relative contributions of each regressor to the total variance in composite RNA expression explained by the resultant regression model.^21^ Stroke remained significantly associated with the composite RNA expression levels of the top 10 genes after accounting for all potentially confounding factors included in the model (Figure 3a), and was responsible for a majority of the explained variance (77.9%, Figure 3b). In terms of potentially confounding factors, both antihypertensive status and anticoagulant status were significantly associated with the composite RNA expression levels of the top 10 genes after accounting for all other regressors (Figure 3a); however, these associations only accounted for a small amount of the variance in composite RNA expression explained by the model (6.5% and 4.5%, respectively, Figure 3b). Results of this multiple regression analysis were supported by the results of a more traditional logistic regression analysis in which the composite RNA expression levels of the top 10 genes were identified as the only significant predictor of stroke when considering the same potentially confounding covariates (Supplementary Table 2). Taken as a whole, these findings suggest that the pattern of differential expression observed across the top 10 genes between groups is highly associated with stroke independently of the assessed potential confounding factors. Although these findings do suggest that antihypertensive status and anticoagulant status may influence the expression levels of the top 10 genes, the effect of this influence on expression levels is likely minimal relative to the effect of stroke, and intergroup differences in these factors were likely not significant drivers of the selection of these genes by GA/kNN.

**Figure 4:**
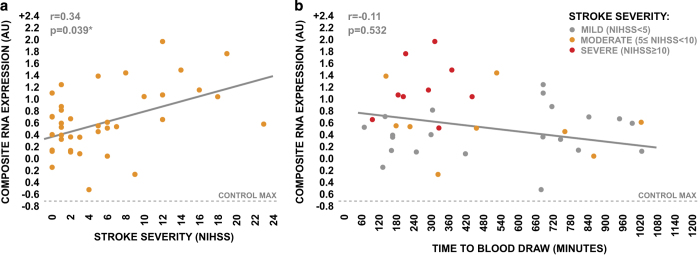
Influence of stroke severity and time to draw blood draw on the coordinate expression levels of the top-ranked genes in discovery cohort AIS patients. (**a**) Relationship between stroke severity, as assessed by NIHSS, and composite RNA expression levels of the top 10 genes in discovery cohort AIS patients. (**b**) Relationship between time from symptom onset to blood draw and composite RNA expression levels of the top 10 genes in discovery cohort AIS patients, with indication of stroke severity. Strength of correlations was tested via Spearman’s rho.

We next used this composite RNA expression variable to examine the potential influence of stroke severity and time to blood draw on the pattern of gene expression observed across the top 10 genes. The composite RNA expression levels of the top 10 genes displayed a significant positive association with stroke severity as assessed by the NIHSS (Figure 4a), suggesting that the expression levels of the top 10 genes are likely directly responsive to stroke pathology. We observed a weak nonsignificant negative relationship between the composite RNA expression levels of the top 10 genes and the time from symptom onset to blood draw (Figure 4b). However, this negative relationship was likely driven by the influence of stroke severity, given that the composite expression levels of these genes were positively associated with stroke severity, and patients undergoing more severe strokes generally presented to the emergency department earlier than patients undergoing less severe strokes (Figure 4b). Collectively, these observations suggest that the stroke-induced differential expression of the top 10 genes may have additional utility for the stratification of stroke severity, and is relatively temporally stable during the acute phase of care.

### Validation cohort

We then tested the diagnostic ability of gene expression pattern identified in the discovery cohort in an independent validation cohort enroled via a second geographically and socioeconomically distinct clinical site (see Materials and methods section). This validation cohort included an additional 39 AIS patients along with two different control groups, one consisting of 30 neurologically asymptomatic controls and the other consisting of 20 acute stroke mimics. Like in the discovery cohort, AIS patients were older than neurologically asymptomatic controls; however, AIS patients and asymptomatic controls were better matched in terms of the prevalence of comorbidities and CVD risk factors (Table 2). AIS patients were also significantly older than stroke mimics, however, extremely well matched in terms of all other clinical and demographic characteristics (Table 2).

**Figure 5:**
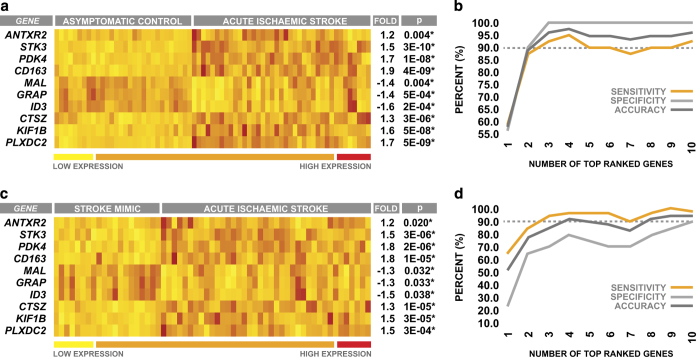
Differential expression and discriminatory ability of top-ranked genes within the validation cohort. (**a**) Peripheral blood differential expression of the top 10 genes between validation cohort neurologically asymptomatic controls and AIS patients. (**b**) Combined ability of the expression levels of the top 10 genes to discriminate between neurologically asymptomatic controls and AIS patients. (**c**) Peripheral blood differential expression of the top 10 genes between acute stroke mimics and AIS patients. (**d**) Combined ability of the expression levels of the top 10 genes to discriminate between acute stroke mimics and AIS patients. All gene expression values are reported as fold change relative to control. Statistical significance of intergroup differences in gene expression was determined via two-sample two-way *t*-test, and *P*-values were corrected to account for multiple comparisons via Holm's Bonferroni method.

Peripheral blood samples were once again obtained from patients at emergency department admission, and the expression levels of the top 10 genes identified by GA/kNN in the discovery cohort were measured via qRT-PCR. The overall pattern of differential expression between AIS patients and asymptomatic controls observed across the top 10 genes in the discovery cohort was also seen when comparing AIS patients and asymptomatic controls in the validation cohort (Figure 5a). The strong ability of the top 10 genes to differentiate between stroke patients and asymptomatic controls in the discovery cohort using kNN was also recapitulated in the validation cohort; the expression levels of the top 10 genes used in combination were able to classify 95.6% of subjects correctly with a sensitivity of 92.3% and a specificity of 100% (Figure 5b).

When comparing AIS patients to stroke mimics, the overall pattern of differential expression observed across the top 10 genes was identical to that observed when comparing AIS patients with asymptomatic controls; however, the magnitude of these expression differences was smaller in the case of several genes (Figure 5c). Despite this reduction in the magnitude of differential expression, the expression levels of the top 10 genes used in combination were still able to accurately discriminate between AIS patients and stroke mimics, classifying 94.9% of subjects correctly with a sensitivity of 97.4% and a specificity of 90.0% (Figure 5d). However, it is important to note that it was evident that all 10 genes were required to achieve high levels of diagnostic accuracy when comparing AIS patients with stroke mimics (Figure 5d), whereas similar levels of accuracy could be achieved with as few as the top four markers when comparing AIS patients with neurologically asymptomatic controls in both the discovery cohort (Figure 1b) and the validation cohort (Figure 5b). Despite this, the collective validation cohort results supported those of the discovery cohort, and provide further evidence that the top 10 markers selected by GA/kNN have high potential performance for identification of AIS.

## Discussion

The primary objective of this study was to apply the GA/kNN approach to identify a pattern of gene expression in peripheral blood that could potentially be optimised to identify AIS in the acute phase of care. The 10 transcriptional markers identified by GA/kNN in our analysis proved robust in their combined ability to differentiate between AIS patients and controls in both the discovery cohort and the independent validation cohort; not only did these markers display levels of diagnostic accuracy that exceed those reported in a majority of previous stroke biomarker studies, they also demonstrated characteristics that suggest they have the potential to be clinically useful. Besides having diagnostic utility, some of the markers identified in this study may represent viable therapeutic targets in the context of stroke immunopathology.

With regards to the countless number of peripheral blood biomarker explorations that have been performed to date, to our knowledge, only one prior investigation has reported similar levels of diagnostic accuracy to those which we observed in this study in terms discriminating between stroke patients and clinically relevant control populations. Dambinova *et al*.^22^ recently reported that plasma levels of brain-derived NR2 peptide, a degradation product of *N*-methyl-D-aspartate receptor cleavage, could be used to differentiate between stroke patients and a combination of acute stroke mimics and neurologically asymptomatic controls with 92% sensitivity and 96% specificity.^22^ However, a majority of blood samples in this prior study were obtained between 24 and 72 h post-symptom onset, and it is currently unknown whether NR2 peptide would exhibit an equivalent level of diagnostic performance early in the acute phase of care. The 10-marker panel identified in our analysis was tested earlier in the progression of pathology, and thus exhibits an obvious advantage in that they has the potential to provide actionable diagnostic information at an early enough time point to influence critical triage decisions that has an impact on outcome.

The 10-marker panel identified in our analysis displayed several favourable characteristics that could make it well suited for identification of ischaemic stroke in the acute care setting. Most notably, the pattern of differential expression we observed between AIS patients and controls appeared to be relatively temporally stable. This is of clinical relevance from the standpoint that it is well established that acute stroke patients tend to arrive to the emergency department in two waves, the first within 4 h from symptom onset (typically patients with more severe overt symptoms), and the second more than 8 h from symptom onset (typically patients with milder symptoms).^23^ For this reason, a potential diagnostic for identification of acute stroke needs to be diagnostically robust across a wide time window with regards to the progression of stroke pathology. Another diagnostically beneficial characteristic we observed was that the stroke-associated pattern of expression across these 10 markers was positively correlated with the NIHSS. Thus, these markers may have utility in stratifying injury severity, information that is commonly considered when making decisions regarding the prescription of interventional treatment.^4^ These characteristics, along with the fact that we observed levels of sensitivity and specificity, which well exceed those achievable via the tools currently available to clinicians for the identification of stroke during acute triage, suggest that the 10-marker panel identified in our analysis has legitimate potential for future clinical implementation.

Besides having diagnostic utility, some of the markers identified in this study may represent potential therapeutic targets in the context of stroke immunopathology. Perhaps, the most interesting of these markers from this standpoint is *CD163*. It is well established that stroke induces a state of peripheral adaptive immune suppression characterised by a limited capacity of lymphoid cells to respond to antigen.^24,25^ This suppressed adaptive immune state leaves patients highly susceptible to post-stroke infection,^26^ which is the leading cause of death in the post-acute phase of care.^27^ *CD163* encodes for a protein known as cluster of differentiation 163 (CD163), a membrane-bound scavenger receptor for extracellular haemoglobin, which is predominantly expressed on immune populations of myeloid lineage.^28,29^ Mature CD163 is known to undergo ectodomain shedding to generate a soluble truncated peptide (sCD163), which has been shown in multiple studies to directly interact with lymphocytes and inhibit antigen-mediated activation.^30–32^ Interestingly, we observed elevated RNA expression levels of CD163 in the peripheral blood of AIS patients; it is possible that CD163 expression is increased in the innate peripheral immune system in response to stroke-induced increases in circulating free haemoglobin,^33^ subsequently driving an increase in levels of circulating sCD163, which act to suppress lymphocyte activation. In support of this hypothesis, unpublished preliminary data from our laboratory suggest that plasma levels of sCD163 are elevated in AIS patients during the acute phase of care, and are positively correlated with RNA expression levels of CD163 in whole blood. Ongoing work in our laboratory is aimed at characterising the relationship between peripheral-blood sCD163 levels and stroke-induced adaptive immune dysfunction, as CD163 may be therapeutically targetable as a means of rescuing adaptive immune responsiveness following stroke.

In addition to *CD163*, the markers identified in this study included several other genes that may be pathologically relevant within the context of the stroke-induced peripheral immune response. We observed downregulated expression levels of *MAL* and *GRAP* in the peripheral blood of AIS patients; both genes encode proteins that are critically involved in T-cell receptor activation and signal transduction.^34,35^ Furthermore, AIS patients exhibited elevated expression levels of *STK3*, a gene encoding a seine threonine kinase involved in pro-apoptotic signal transduction^36,37^ and suppression of lymphocyte proliferation.^38^ Taken as a whole, the differential regulation we observed across these genes is consistent with suppressed adaptive immune state induced in response to stroke, and may be mechanistically involved in blunting the responsiveness of the adaptive immune system following ischaemic brain injury. Conversely, two of the markers identified as being upregulated in the peripheral blood of AIS patients in this study, *KIF1B* and *ANTXR2*, may be mechanistically involved in the innate immune response to ischaemic insult. It is well established that stroke induces robust recruitment of myeloid-derived innate immune populations such as neutrophils and monocytes from the peripheral blood into the brain parenchyma;^39,40^ both genes encode proteins that have been shown to have a role in cellular adhesion and migration,^41–44^ and thus may be mechanistically involved in this process.

Collectively, the findings reported here are exciting; however, it is important to note that this study was not without limitations. Perhaps, most notably was the fact that AIS patients and neurologically asymptomatic controls in our discovery cohort were not well matched with regards to several clinical and demographic characteristics; thus, intergroup differences in these factors had the potential to confound the selection of stroke-specific genes in our GA/kNN analysis. To account for this possible limitation, we utilised a relatively high termination cutoff for optimal solution selection; under these conditions, a confounding factor would have to be almost ubiquitously present in one group, and nearly ubiquitously absent in the other, for it to influence the selection of candidate genes. The results of our multiple regression analysis suggest that this strategy was largely successful; however, they did infer that medication status may influence the expression of the candidate genes. Despite this, the 10 candidate genes were still able to demonstrate high levels of diagnostic accuracy when discriminating between groups that were better matched in terms of these factors in the validation cohort.

Taken as a whole, the results of this preliminary study demonstrate that a highly accurate RNA-based companion diagnostic for AIS is plausible using a relatively small number of markers, and also highlight the potential power of machine-learning approaches for biomarker discovery in the realm of CVD. The 10 transcriptional biomarkers identified in this study displayed levels of diagnostic performance that well exceed those reported in a majority of previous stroke biomarker investigations, as well as several characteristics that suggest that they may have true clinical utility for identification of ischaemic stroke during the acute phase of care. Furthermore, future exploration of these markers may reveal novel mechanisms that underlie the peripheral immune response to stroke, and lead to novel therapeutic targets in the context of stroke-induced immunopathology. Owing to the robust results of this preliminary analysis, the 10 transcriptional biomarkers identified in this study warrant further evaluation to determine their true clinical efficacy.

## Materials and methods

### Discovery cohort patients

Acute ischaemic stroke patients and neurologically asymptomatic controls were recruited at Suburban Hospital, Bethesda, MD, USA, which serves an upper-class metro area bordering Washington DC. AIS cases were of mixed aetiology, and diagnosis was confirmed using magnetic resonance imaging according to the established criteria for diagnosis of acute ischaemic cerebrovascular syndrome.^45^ The median time from symptom onset to blood draw was 5.3 h, as determined by the time the patient was last known to be free of AIS symptoms. In the case of patients who received thrombolytic therapy, blood samples were collected before the administration of recombinant tissue plasminogen activator. Injury severity was determined according to NIHSS at the time of blood draw. Control subjects were deemed neurologically normal by a trained neurologist at the time of enrolment. Demographic information was collected from either the subject or significant other by a trained clinician. All procedures were approved by the institutional review boards of the National Institute of Neurological Disorders/National Institute on Aging at the National Institutes of Health and Suburban Hospital. Written informed consent was obtained from all subjects or their authorised representatives before any study procedures.

### Blood collection and RNA extraction

Peripheral whole-blood samples were collected via PAXgene RNA tubes (Qiagen, Valencia, CA, USA) and stored at −80 °C until RNA extraction. Total RNA was extracted via the PreAnalytiX PAXgene blood RNA Kit (Qiagen) and automated using the QIAcube System (Qiagen). Quantity and purity of isolated RNA was determined via spectrophotometry (NanoDrop, Thermo Scientific, Waltham, MA, USA). Quality of RNA was confirmed by chip capillary electrophoresis (Agilent 2100 Bioanalyzer, Agilent Technologies, Santa Clara, CA, USA).

### RNA amplification and microarray

RNA was amplified and biotinylated using the TotalPrep RNA Amplification Kit (Applied Biosystems, Grand Island, NY, USA). Samples were hybridised to HumanRef-8 expression bead chips (Illumina, San Diego, CA, USA) containing 25,000 unique probes and scanned using the Illumina BeadStation. Raw probe intensities were background-subtracted, quantile-normalised and then summarised at the gene level using Illumina GenomeStudio. Sample labelling, hybridisation and scanning were performed per standard Illumina protocols. Raw data are assessable through the National Center for Biotechnology Information Gene Expression Omnibus via accession number GSE16561.

### GA/kNN analysis

Normalised microarray data were filtered based on absolute fold difference between stroke and control; genes exhibiting a greater than 1.7 absolute fold difference in expression between AIS and control were retained for analysis. Filtered gene expression data were *z*-transformed and GA/kNN analysis was performed using C source code developed by Li *et al.* ^20^ compiled in Linux Mint. Two thousand near-optimal solutions were collected per sample using five nearest neighbours, majority rule, a chromosome length of five and a termination cutoff of 0.97. Leave-one-out cross-validation was performed using the top 50 ranked genes. The top 50 genes were tested against random gene combinations, which were selected using the R sample() function (R 2.14, R Project for Statistical Computing).

### Validation cohort patients

AIS patients, acute stroke mimics and neurologically asymptomatic controls were recruited at Ruby Memorial Hospital, Morgantown, WV, USA, which serves an impoverished rural region of West Virginia that displays some of the highest CVD rates in the nation.^1^ As with the discovery cohort, AIS cases were of mixed aetiology, and diagnosis was confirmed via neuroradiological imaging. Patients admitted to the emergency department as suspected strokes based on the overt presentation of stroke-like symptoms, but receiving a negative diagnosis for stroke upon imaging according to the established acute ischaemic cerebrovascular syndrome diagnostic criteria,^45^ were identified as acute stroke mimics. Discharge diagnoses of stroke mimics included cases of seizures, complex migraines and other conditions, which induce neurological symptoms such as hypertensive encephalopathy. The median time from symptom onset to blood draw was 4.6 h and all blood was sampled before the administration of recombinant tissue plasminogen activator. Assessment of injury severity, screening of neurologically asymptomatic controls and collection of demographic information were performed in an identical manner. All procedures were approved by the institutional review boards of West Virginia University and Ruby Memorial Hospital. Written informed consent was obtained from all subjects or their authorised representatives before study procedures.

### Quantitative reverse transcription PCR

Complementary DNA was generated from purified RNA using the Applied Biosystems high-capacity reverse transcription kit. For qPCR, target sequences were amplified from 10 ng of complementary DNA input using sequence-specific primers (Supplementary Table 3) and detected via SYBR green (PowerSYBR, Thermo Fisher, Waltham, MA, USA) on the RotorGeneQ (Qiagen). Raw amplification plots were background-corrected and CT values were generated via the RotorGeneQ software package. All reactions were performed in triplicate. Transcripts of *B2M*, *PPIB* and *ACTB* were amplified as references, and normalisation was performed using the NORMAgene data-driven normalisation algorithm.^46^

### Statistical analysis

Parametric statistical analysis was performed using SPSS (IBM, Chicago, IL, USA) in combination with R 2.14 via the SPSS R integration plug-in. *χ* ^2^-tests were used for comparison of dichotomous variables, whereas Student's *t*-tests were used for comparison of continuous variables. Spearman’s rho was used to assess the strength of correlational relationships. For multiple regression analysis, variance decomposition was performed using the relaimpo R package.^21^ Penalised logistic regression was performed using the logistf R package.^47^ The level of significance was established at 0.05 for all parametric statistical testing. In the cases of multiple comparisons, *P*-values were adjusted using Holm’s Bonferonni method.^48^

## Figures and Tables

**Table 1:** Discovery cohort clinical and demographic characteristics

	*Control (*n*=24)*	*AIS (*n*=39)*	*Statistic (df)*	P
Age (mean±s.d.)	59.9±9.7	73.1±14.0	*t*=−4.40 (61)	>0.001*
Female *n* (%)	14 (58.3)	22 (56.4)	*χ* ^2^=0.12 (1)	0.731
NIHSS (mean±s.d.)	0±0.0	5.3±6.4	*t*=5.17 (38)	>0.001*
Family history of stroke *n *(%)	4 (16.7)	15 (38.5)	*χ* ^2^=7.02 (1)	0.008*
Hypertension *n *(%)	7 (29.2)	25 (64.1)	*χ* ^2^=11.2 (1)	0.001*
Dyslipidaemia *n* (%)	0 (0.00)	18 (46.2)	*χ* ^2^=15.5 (1)	>0.001*
Diabetes *n *(%)	2 (8.30)	11 (28.2)	*χ* ^2^=3.58 (1)	0.058
Previous stroke *n* (%)	2 (8.30)	6 (15.4)	*χ* ^2^=0.67 (1)	0.414
Atrial fibrillation *n* (%)	0 (0.00)	6 (15.4)	*χ* ^2^=4.08 (1)	0.043*
Myocardial infarction *n* (%)	0 (0.00)	6 (15.4)	*χ* ^2^=4.08 (1)	0.043*
Hypertension medication *n* (%)	8 (33.3)	29 (74.4)	*χ* ^2^=10.3 (1)	0.001*
Diabetes medication *n* (%)	1 (4.20)	7 (17.9)	*χ* ^2^=2.55 (1)	0.111
Cholesterol medication *n *(%)	5 (20.8)	17 (43.6)	*χ* ^2^=3.39 (1)	0.066
Anticoagulant or antiplatelet *n *(%)	1 (4.20)	20 (51.3)	*χ* ^2^=14.9 (1)	>0.001*
rtPA *n *(%)	0 (0.00)	9 (23.1)	*χ* ^2^=6.46 (1)	0.011*
Current smoker *n* (%)	2 (8.30)	2 (5.13)	*χ* ^2^=0.26 (1)	0.612

Abbreviations: AIS, acute ischaemic stroke; df, degrees of freedom; NIHSS, National Institutes of Health stroke scale; rtPA, recombinant tissue plasminogen activator.

*Indicates statistically significant values.

**Table 2:** Validation cohort clinical and demographic characteristics

	*Asymptomatic control versus AIS*				*Mimic versus AIS*			
	*Control (*n*=30)*	*AIS (*n*=39)*	*Statistic (df)*	P	*Mimic (*n*=20)*	*AIS (*n*=39)*	*Statistic (df)*	P
Age (mean±s.d.)	51.5±14.3	73.1±13.3	*t*=−6.41 (67)	>0.001*	58.0±17.0	73.1±13.3	*t*=−3.78 (57)	>0.001*
Female *n* (%)	25 (83.3)	25 (64.1)	*χ* ^2^=3.14 (1)	0.076	9 (45.0)	25 (64.1)	*χ* ^2^=1.98 (1)	0.159
NIHSS (mean±s.d.)	0.0±0.0	8.6±7.5	*t*=7.16 (38)	>0.001*	4.7±4.9	8.6±7.5	*t*=−2.11 (57)	0.041*
Family history of stroke *n *(%)	16 (53.3)	15 (38.5)	*χ* ^2^=1.52 (1)	0.213	5 (25.0)	15 (38.5)	*χ* ^2^=1.07 (1)	0.301
Hypertension *n *(%)	17 (56.7)	32 (82.1)	*χ* ^2^=5.31 (1)	0.021*	17 (85.0)	32 (82.1)	*χ* ^2^=0.08 (1)	0.775
Dyslipidaemia *n* (%)	11 (36.7)	16 (41.0)	*χ* ^2^=0.14 (1)	0.713	13 (65.0)	16 (41.0)	*χ* ^2^=3.08 (1)	0.081
Diabetes *n* (%)	2 (6.70)	8 (20.5)	*χ* ^2^=2.62 (1)	0.105	7 (35.0)	8 (20.5)	*χ* ^2^=1.46 (1)	0.226
Previous stroke *n* (%)	1 (3.30)	7 (17.9)	*χ* ^2^=3.53 (1)	0.061	5 (25.0)	7 (17.9)	*χ* ^2^=0.52 (1)	0.524
Atrial fibrillation *n* (%)	0 (0.00)	13 (33.3)	*χ* ^2^=12.3 (1)	>0.001*	3 (15.0)	13 (33.3)	*χ* ^2^=2.25 (1)	0.134
Myocardial infarction *n *(%)	0 (0.00)	11 (28.2)	*χ* ^2^=10.0 (1)	0.002*	6 (30.0)	11 (28.2)	*χ* ^2^=0.02 (1)	0.885
Hypertension medication *n* (%)	15 (50.0)	27 (69.2)	*χ* ^2^=2.63 (1)	0.105	16 (80.0)	27 (69.2)	*χ* ^2^=0.78 (1)	0.378
Diabetes medication *n *(%)	2 (6.70)	8 (20.5)	*χ* ^2^=2.62 (1)	0.105	6 (30.0)	8 (20.5)	*χ* ^2^=0.66 (1)	0.418
Cholesterol medication *n* (%)	7 (23.3)	14 (35.9)	*χ* ^2^=1.26 (1)	0.261	12 (60.0)	14 (35.9)	*χ* ^2^=3.12 (1)	0.078
Anticoagulant or antiplatelet *n* (%)	1 (3.30)	23 (59.0)	*χ* ^2^=23.1 (1)	>0.001*	12 (60.0)	23 (59.0)	*χ* ^2^=0.01 (1)	0.939
rtPA *n *(%)	0 (0.00)	13 (33.3)	*χ* ^2^=12.3 (1)	>0.001*	0 (0.00)	13 (33.3)	*χ* ^2^=8.55 (1)	0.004*
Current smoker *n* (%)	1 (3.30)	9 (23.1)	*χ* ^2^=5.33 (1)	0.021*	2 (10.0)	9 (23.1)	*χ* ^2^=1.49 (1)	0.222

Abbreviations: AIS, acute ischaemic stroke; df, degrees of freedom; NIHSS, National Institutes of Health stroke scale; rtPA, recombinant tissue plasminogen activator.

*Indicates statistically significant values.
