# Supplementary material for: Machine-learning approach identifies a pattern of gene expression in peripheral blood that can accurately detect ischaemic stroke
Source: NPJ Genom Med. 2016 Nov 30;1:16038–. doi: 10.1038/npjgenmed.2016.38 (PMC5685316; doi:10.1038/npjgenmed.2016.38)
Supplement: Supplementary Figure 1 [file npjgenmed201638-s1.pdf]

SUPPLEMENTAL FIGURE 1.

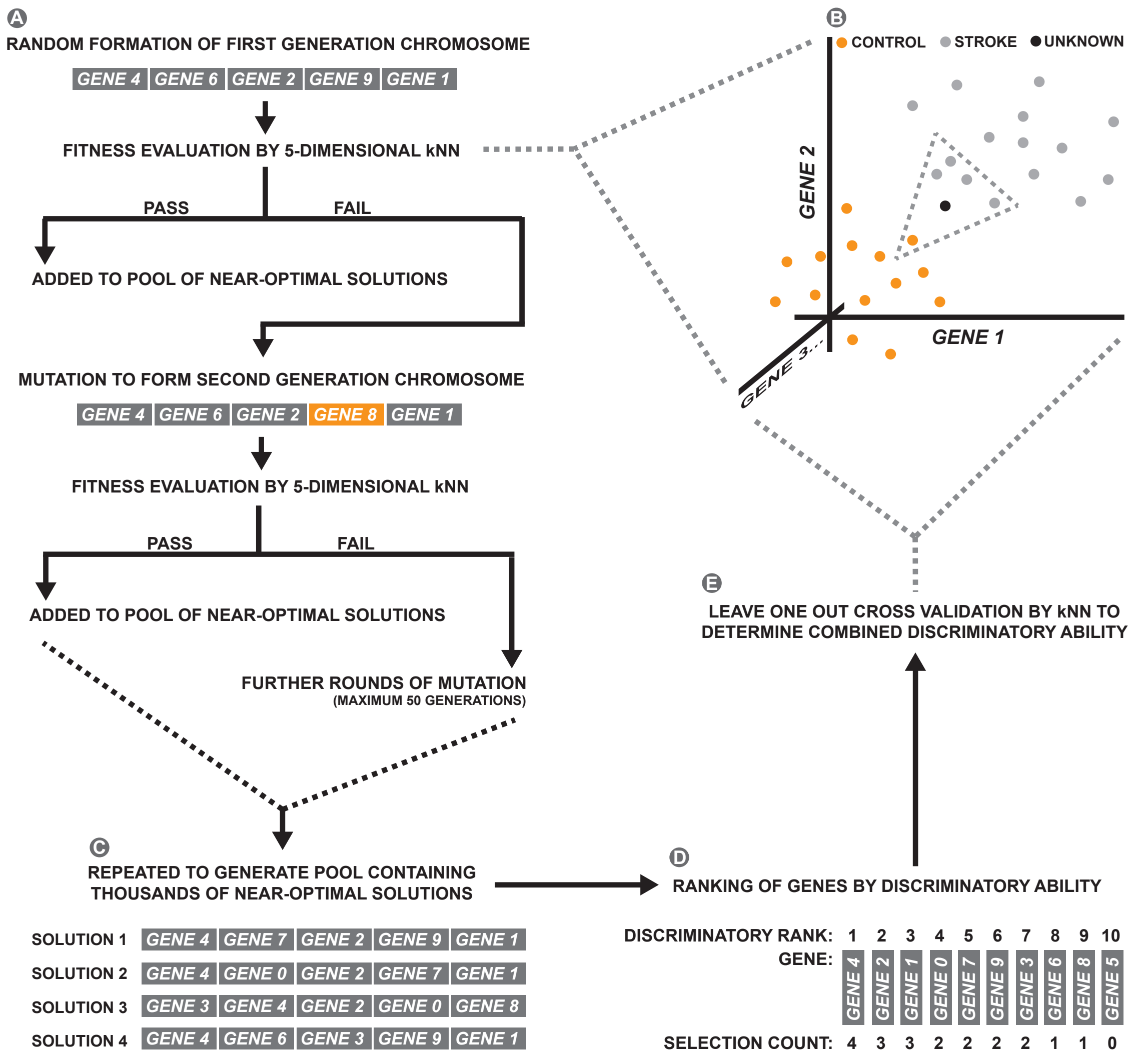

GA/kNN paradigm used for the selection of transcriptional biomarkers from high-dimensional expression data. (A) A first generation chromosome is generated via random selection from the total pool of gene expression data. (B) The ability of the first generation chromosome to discriminate between groups is evaluated using kNN. (A) If the chromosome passes evaluation, it is added to the pool of near-optimal solutions. If the chromosome fails evaluation, it undergoes mutation and re-evaluation until a near-optimal solution is found. (C) This search is repeated to generate a pool containing hundreds of thousands of near-optimal solutions. (D) Genes are then ranked based on the number of times they appear in the near-optimal solution pool. (E) The combined discriminatory ability of the top ranked genes is then tested in leave one out cross validation via kNN.
