# Supplementary material for: Machine-learning approach identifies a pattern of gene expression in peripheral blood that can accurately detect ischaemic stroke
Source: NPJ Genom Med. 2016 Nov 30;1:16038–. doi: 10.1038/npjgenmed.2016.38 (PMC5685316; doi:10.1038/npjgenmed.2016.38)
Supplement: Supplementary Table 1 [file npjgenmed201638-s2.pdf]

SUPPLEMENTAL TABLE 1.

A

r (p)

	MAL	*GRAP*	*ID3*	*PLXDC2*	*KIF1B*	*CD163*	*CTSZ*	*PDK4*	*STK3*
GRAP	0.93 (<0.001)								
ID3	0.85 (<0.001)	0.88 (<0.001)							
PLXDC2	-0.72 (<0.001)	-0.80 (<0.001)	-0.82 (<0.001)						
KIF1B	-0.65 (<0.001)	-0.71 (<0.001)	-0.69 (<0.001)	0.81 (<0.001)					
CD163	-0.76 (<0.001)	-0.76 (<0.001)	-0.79 (<0.001)	0.83 (<0.001)	0.70 (<0.001)				
CTSZ	-0.43 (<0.001)	-0.46 (<0.001)	-0.46 (<0.001)	0.57 (<0.001)	0.54 (<0.001)	0.58 (<0.001)			
PDK4	-0.59 (<0.001)	-0.63 (<0.001)	-0.52 (<0.001)	0.51 (<0.001)	0.54 (<0.001)	0.59 (<0.001)	0.38 (0.001)		
STK3	-0.67 (<0.001)	-0.72 (<0.001)	-0.64 (<0.001)	0.74 (<0.001)	0.78 (<0.001)	0.79 (<0.001)	0.59 (<0.001)	0.75 (<0.001)	
ANTXR2	-0.51 (<0.001)	-0.56 (<0.001)	-0.60 (<0.001)	0.77 (<0.001)	0.78 (<0.001)	0.63 (<0.001)	0.58 (<0.001)	0.41 (<0.001)	0.58 (<0.001)

KMO: 0.88 BARTLETTS SPHERICITY: p<0.001

PC1:
70% VARIANCE
λ=6.99

B

	LOADINGS	COEFFICENT
MAL	-0.86	-0.12
GRAP	-0.91	-0.13
ID3	-0.88	-0.13
PLXDC2	0.92	0.13
KIF1B	0.87	0.12
CD163	0.90	0.13
CTSZ	0.66	0.09
PDK4	0.70	0.10
STK3	0.87	0.12
ANTXR2	0.76	0.11

COMPOSITE RNA EXPRESSION

Use of principal components analysis for generation of a composite RNA expression variable summarizing the coordinate expression levels of the top ten genes. (A) Correlation matrix depicting the correlations between the expression levels of the top 10 genes identified by GA/kNN in the discovery cohort. (B) Principal component (PC) loadings and coefficients used for generation of the composite RNA expression variable.
