# Supplementary material for: Machine-learning approach identifies a pattern of gene expression in peripheral blood that can accurately detect ischaemic stroke
Source: NPJ Genom Med. 2016 Nov 30;1:16038–. doi: 10.1038/npjgenmed.2016.38 (PMC5685316; doi:10.1038/npjgenmed.2016.38)
Supplement: Supplementary Table 2 [file npjgenmed201638-s3.pdf]

SUPPLEMENTAL TABLE 2.

Regression Summary: Likelihood Ratio Test=60.0, df=8, p=5E-10 *

	B	Std Error	χ^2	p
Intercept	-2.23	3.39	0.24	0.63
**Composite RNA Expression**	3.02	0.85	24.83	6E-7 *
**Age**	0.05	0.06	0.45	0.51
**Hypertension**	0.28	1.62	0.01	0.91
**Atrial Fibrillation**	-1.31	2.42	0.21	0.65
**Myocardial Infarction**	-1.27	2.13	0.20	0.66
**Dyslipidemia**	0.90	1.81	0.14	0.71
**Hypertension Medication**	-1.17	1.55	0.43	0.51
**Anticoagulant/Antiplatelet**	1.69	1.37	0.88	0.35

Influence of potentially confounding clinical and demographic characteristics on the predictive power of the top ten genes. Firth's penalized logistic regression was performed with stroke as the dependent variable and composite RNA expression, age, anticoagulant status, hypertension, antihypertension medication status, dyslipidemia, history of myocardial infarction, and history of atrial fibrillation as regressors. In the resultant model, composite RNA expression remained a highly significant predictor of stroke after considering all other regressors. Furthermore, none of the potentially confounding factors were significant predictors of stroke in the model.
