# Supplementary material for: Machine-learning approach identifies a pattern of gene expression in peripheral blood that can accurately detect ischaemic stroke
Source: NPJ Genom Med. 2016 Nov 30;1:16038–. doi: 10.1038/npjgenmed.2016.38 (PMC5685316; doi:10.1038/npjgenmed.2016.38)
Supplement: Supplementary Table 3 [file npjgenmed201638-s4.pdf]

SUPPLEMENTAL TABLE 3. Primers and thermocycling conditions used for qRT-PCR.

GENE	TRANSCRIPTS[1]	PRIMERS (5' to 3')[2]		PRODUCT (bp)
ANTXR2	NM_058172.5<br>NM_001145794.1	**FOR:**<br>**REV:**	GATCTCTACTTCGTCCTGGACA<br>AAATCTCTCCGCAAGTTGCTG	**90**
STK3	NM_006281.3<br>XM_011517258.1<br>XM_011517255.1<br>XM_011517254.1<br>XM_011517253.1<br>XM_011517252.1<br>XM_011517251.1<br>XM_011517250.1<br>XM_011517249.1<br>XM_011517247.1<br>NM_001256312.1<br>NM_001256313.1	**FOR:**<br>**REV:**	CGATGTTGGAATCCGACTTGG<br>GTCTTTGTACTTGTGGTGAGGTT	**105**
PDK4	NM_002612.3	**FOR:**<br>**REV:**	GACCCAGTCACCAATCAAAATCT<br>GGTTCATCAGCATCCGAGTAGA	**82**
CD163	NM_004244.5<br>XM_005253529.3<br>XM_005253528.3<br>NM_203416.3	**FOR:**<br>**REV:**	GCGGGAGAGTGGAAGTGAAAG<br>GTTACAAATCACAGAGACCGCT	**89**
MAL	NM_002371.3<br>NM_022439.2	**FOR:**<br>**REV:**	GCCCTCTTTTACCTCAGCG<br>GCAATGTTTTCATGGTAGTGCCT	**95**
GRAP	NM_006613.3	**FOR:**<br>**REV:**	AGCCCTTGCTCAAGTCACC<br>CGTAACTCCGTGGGAAGAAGC	**180**
ID3	NM_002167.4	**FOR:**<br>**REV:**	GAGAGGCACTCAGCTTAGCC<br>TCCTTTTGTCGTTGGAGATGAC	**170**
CTSZ	NM_001336.3	**FOR:**<br>**REV:**	CAGCGGATCTGCCCAAGAG<br>CGATGACGTTCTGCACGGA	**198**
PLXDC2	NM_032812.8<br>XM_011519750.1	**FOR:**<br>**REV:**	ACTCAGATCGAGGAGGATACAGA<br>CCGGCTGGCAGAATCAGATG	**75**
KIF1B	NM_015074.3<br>NM_183416.3	**FOR:**<br>**REV:**	AAACAAGGGTAATTTGCGTGTGC<br>GTAACTGCCAACTTGGACAGAT	**78**
PPIB	NM_000942.4	**FOR:**<br>**REV:**	AAGTCACCGTCAAGGTGTATTTT<br>TGCTGTTTTTGTAGCCAAATCCT	**153**
B2M	NM_004048.2<br>XM_006725182.2<br>XM_005254549.2	**FOR:**<br>**REV:**	GAGGCTATCCAGCGTACTCCA<br>CGGCAGGCATACTCATCTTTT	**248**
ACTB	NM_001101.3<br>XM_006715764.1	**FOR:**<br>**REV:**	CATGTACGTTGCTATCCAGGC<br>CTCCTTAATGTCACGCACGAT	**250**

[1]Listed by NCBI accession number

[2]All targets were amplified for 40 cycles of 95ºC (15s) / 60ºC (60s)
